# Self-managed abortion: a constellation of actors, a cacophony of laws?

**DOI:** 10.1080/26410397.2021.1899764

**Published:** 2021-03-25

**Authors:** Lucía Berro Pizzarossa, Rishita Nandagiri

**Affiliations:** a Postdoctoral Fellow, O'Neill Institute for National and Global Health Law, Georgetown University Law Center, Washington, DC, USA; b ESRC Postdoctoral Fellow, Department of Methodology, London School of Economics and Political Science, London, UK

**Keywords:** self-managed abortion, abortion access, abortion laws, abortion networks, medication abortion

Self-managed abortion (SMA) is not a new phenomenon but occurs across histories^1^ and social and legal contexts,^2^ utilising a range of methods.^1^ SMA is broadly understood as actions or activities undertaken by a pregnant individual to end a pregnancy outside of clinical settings, but there is considerable debate around how SMA is understood. These debates are underpinned by a range of approaches, politics and standpoints. Language use also varies (e.g. self-administered or self-care), reflecting the types of technologies or individuals involved.

The steady increase in the use of medical abortion (MA) drugs – misoprostol and mifepristone – has enabled safer self-management and self-use, centring autonomy, privacy and confidentiality, while also contributing to the reduction of abortion-related morbidity and mortality globally.^3,4^ MA has increasingly been included as an element of sexual and reproductive health interventions and is gaining greater consideration within notions of self-care.^5^ The advent of telehealth and the growing network of organisations supporting safe self-use has fundamentally altered the abortion landscape.^6–9^ This is also evident in the temporary shift from some governments during the COVID-19 pandemic, allowing abortion via telemedicine ^9–11^ or “pills by post”.^12^ Medical societies and organisations^13^ have also called for a similar shift to provision via telemedicine.^14^ We understand “telemedicine” as the provision of remote clinical services through formal systems, while “telehealth” covers a broad range of health activities (e.g. health promotion activities) that are provided remotely through technology and other platforms. We acknowledge (and agree with) feminist groups’ disagreement around accompaniment models, information or safe abortion hotlines being classified as “telemedicine”, especially as their approaches directly challenge the medicalisation of abortion.

These shifts in the abortion landscape demonstrate how SMA – through the use of MA – challenges binary conceptualisations of abortion safety,^15^ unsettling heavily medicalised notions of what safe conditions are and who a provider is. By enabling and centring the needs and autonomies of abortion-seekers, SMA reclaims abortion autonomy as a feminist political demand.^16,17^ Yet rather than a solely individual act, pregnant persons’ SMA trajectories are shaped and influenced by a number of actors at different points along their journey. These actors, functioning locally and nationally, as well as transnationally, enable SMA access and provide different types of support.^17^ For example, feminist actors on the ground and the networks procuring pills, disseminating information and providing assistance over the course of people’s abortion trajectories have enabled SMA.^18,19^ They have also, through these efforts, shifted understandings of self-induced abortion from “risky”, “unsafe”, “back alley abortions” to a notion of “self-care” and a reconceptulisation of “safety”.^16^ As governments enact strict anti-abortion laws (e.g. Poland, USA), are unable to provide full-spectrum abortion care (e.g. Ireland), or where overburdened health systems, whether due to the pandemic or chronic underfunding, cannot provide adequate care, it is a range of formal and informal actors – hotlines, feminist networks, accompaniment groups, doulas, pharmacists or other community health workers – who step in to provide care, sometimes across borders.^20^ Providing critical information and support, these actors are made vulnerable and exposed to social and legal risks because of their work around SMA. They experience direct criminalisation and/or risk of imprisonment due to contravention of a related, but not abortion-specific, law (e.g. illegal pharmacy practices) or experience increased surveillance and harassment (e.g. criminalising a mother for procuring MA for her underage daughter), among other threats and risks to their lives and livelihoods.

Understandings of SMA, while continuing to centre the needs of those seeking an abortion, need to expand to account for, acknowledge and consider how these actors and their roles are theorised, supported and understood. These shifts and changes in the abortion landscape are not reflected in the way abortion continues to be legally framed and regulated, requiring a deeper consideration of the ecology of abortion care-seeking and care-provision (including SMA), its contexts and its actors.

Thus, we define self-management of abortion as consisting of a range of individual activities – a multiplicity of behaviours and navigations that surround abortion self-use (e.g. self-sourcing, potentially necessitating (unpaid) leave from work, arranging childcare and management of symptoms or complications, confirmation of abortion), **and** the collective dimension that enables safe self-use through a constellation of actors and interlocutors (e.g. friends, partners, family members, community health intermediaries, pharmacists, activists, non-profit organisations, hotline operators, accompaniment networks, doulas) who undertake a number of activities (e.g. provision of accurate information, sourcing of pills, accompaniment through the process, childcare provision) to support peoples’ SMA trajectories.^16^ In this commentary, while recognising the spectrum of methods and approaches used to self-manage an abortion, we speak largely to SMA using MA and focus on those actors who support womxn (we use “womxn” or “pregnant persons” as transmen and non-binary persons, along with women and girls, need or require abortions).

## A constellation of actors?

Discussions on SMA have largely focused on its potential for womxn’s agencies and realising reproductive freedoms,^21^ shifting away from a medicalised and paternalistic model of abortion care.^22^ This justified individual focus on womxn, however, overlooks the essential collective of actors, processes and communities that surround, enable and support this claiming and enacting of agencies. Some, in this broad constellation of actors, can also work to enact barriers to abortion care and self-management.

This constellation of actors involves persons, groups and collectives working in/formally and il/legally, and at different points in womxn’s trajectories to enable or restrict SMA.^16^ A non-exhaustive mapping would include, starting with the pregnant persons themselves: feminist groups, accompaniment networks, abortion hotlines, full-spectrum doulas, friends, partners or family members, community health workers, websites sharing abortion information, clinic escorts, pharmacists or other friendly providers and many others that we may not have yet considered. It includes anti-abortion groups and actors who attempt to misdirect or remove SMA access. Individuals and groups who may not align themselves with anti-abortion ideologies, but nonetheless hold stigmatising beliefs or are misinformed about abortion, may also further abortion stigma, affecting womxn’s trajectories and experiences including SMA.

At the individual or interpersonal level, evidence shows that social networks are essential to SMA. In India, for example, the majority of abortions are estimated to be medical abortions occurring outside formal health facilities.^23^ Men play an important role in purchasing pills for their partners,^24^ and lay community health intermediaries can offer crucial interventions including information or procuring pills. These may involve some health workers, like pharmacists for example, working outside their legal and professional remit to cater to individual needs.^25^ It may also involve, where restrictions have been enforced, illegal vendors, as observed in Brazil and other countries.^6^ The role of pharmacists and cadres of community health workers in MA has received increasing attention, particularly in contexts where abortion is legal.^26–28^ These task-sharing roles, however, remain largely conceptualised within formal spaces rather than SMA.^25^ This is despite evidence that pharmacists and lay community health intermediaries play important roles in SMA outside of formal spaces, blurring the line between formal and informal abortion service and information provision.^2,24^

At the community and social networks level, the role of feminist networks and organisations in enabling access cannot be overstated. Even before the pandemic, feminist accompaniment networks, abortion hotlines and websites were catering to essential abortion needs ^6,7,29,30^ which have become a lifeline during lockdown.^31^ Staffed by trained workers and volunteers, these support systems often provide information and services where none are otherwise available or accessible including due to legal restrictions.^6,8,29^ Apart from providing crucial counselling and support, they also normalise and validate womxn’s abortion needs and SMA experiences.^29,32^ As exemplified by the *Socorristas* in Argentina, the accompaniment model provides support over the course of the abortion trajectory. Their work also includes supporting self-management of second-trimester abortions,^33^ involving a range of activists and experts from trained telephone operators and pharmacists to collective knowledge-sharing of experts, links with formal care-providers if needed and provision of post-abortion follow-up by trained clinicians. All of these different actors enable self-management, forming that essential constellation of actors who provide information, accompaniment, medication, support, counselling and post-abortion care including referrals for a range of needs (e.g. psychological care, support for survivors of violence). Without them, SMA would likely involve greater levels of risk, be harder to access and womxn might resort to more unsafe methods.

Google searches for abortion pills have more than doubled in the last decade, especially in contexts where abortion is legally restricted.^34^ Abortion websites and those who write the content and keep it updated do not just provide accurate (and often hard-to-find) abortion information,^20^ including on SMA, but are tasked with signposting other necessary resources (especially when things are fast-changing or where government restrictions may make it a target for shutdown), as well as actively identifying and warning against misinformation and proliferation of harmful, fake pills.^35^ They also contend with overzealous moderation of social media sites leading to suspensions^36^ or blocking of websites.^37^ Websites and social media accounts, often run by feminist networks, also offer emotional, legal and practical support for womxn, especially when they may need to safeguard secrecy around abortion for safety or legal reasons. As technology and internet access has improved, feminist groups have also turned to using messaging apps to provide information and support.^38^This too requires training, skills and resources. This existing infrastructure has been a boon during the COVID-19 pandemic, with many womxn accessing help without compromising their personal privacy or safety.^39^ The support provided across these mediums can extend from linking access to care, explaining what symptoms or signs womxn may experience to validating their decisions.^40,41^ This work in enabling safe abortion access exceeds just the provision of information or services alone but contributes to an environment in which womxn can exercise their autonomies fully informed and in a safe manner.

All these actors are exposed to risk (legal, social, physical and emotional, in addition to personal and collective risk) and carry an increased burden of responsibilities in their work to enable SMA. While there have been efforts to consider how formal abortion providers are stigmatised or experience risk,^42^ there is little understanding of the stigma, vulnerabilities and risks this constellation of actors are exposed to. These actors – and the risks they experience and burdens they carry – remain hidden within SMA narratives, despite being crucial players in womxn’s SMA trajectories and experiences.

The constellation also includes anti-abortion actors and groups, from those spreading anti-abortion myths on social media to funded websites deliberately seeking to misdirect abortion-seekers,^43,44^ or using technology to block access through misinformation and lies.^45^ Advertisements may also contain inaccurate or misleading information on abortion^46^ and may, like so-called “crisis pregnancy centres”, co-opt feminist language in their misdirection efforts.^14^ Overlooking these groups and actors within the abortion ecology is dangerous, particularly in light of increased efforts to restrict abortion globally. Even in the midst of a pandemic, a UK anti-abortion group conducted a bogus study to discredit telemedicine,^47^ taking the British Pregnancy Advisory Service to court and losing an appeal against the at-home abortion service. These tactics contribute to the misinformation and stigma around at-home abortions, feeding into the continued medicalisation of abortion and increasing the risk womxn are exposed to. It also stigmatises individuals and groups working on and providing abortion access. Apart from their negative impact on abortion-seekers, such efforts waste feminist groups’ scarce resources and time, especially when fighting spurious accusations that threaten to limit abortion care.

## A cacophony of laws?

Abortion laws, even in their “best” constructions, set burdensome requirements for access.^48^ Even new laws that bring about liberalisation of abortion still propose a model of criminalisation or medicalisation of SMA and as such create vulnerabilities and risks for those engaged in the practice.

Under most current laws, people that self-manage and those who provide them with information, support or accompaniment risk arrest, police harassment or bribery, prosecution and imprisonment. Indeed, laws such as the Uruguayan^49^ or South African^50^ laws that are considered ground-breaking, still criminalise those who self-manage their abortions. These laws are part of the majority of laws that require medical involvement (multiple medical professionals in these two cases) and specifically criminalise those that procure abortions outside of the process set up in the law, while simultaneously placing very burdensome barriers to access.^51^ Criminalising people who self-manage their abortions has no societal purpose nor any benefit for womxn’s health.

In contrast, under the Irish and New Zealand laws (2019 and 2020), it is no longer a crime for a pregnant person to have an abortion, even if they do so outside of the provisions of the Act. Yet, these laws overlook a large proportion of this “constellation of actors”; family members, friends, support networks or doulas that assist in the procurement of an abortion outside of the procedures set by law can still be criminalised for doing so.^52,53^ For example, in Rwanda, womxn who have obtained illegal abortions have been pardoned, but those who aided them continue to serve full sentences.^54^ The criminalisation of those who assist goes well beyond the harms the law aimed to address (coerced abortion). It creates a stand-alone crime (that does not exist for any other medical matter), further exceptionalising abortion when these concerns could easily have been covered by general criminal law and professional regulations.

Additionally, laws and policies create vulnerabilities and risks for those engaged in the practice by overregulating access to medicines or restricting the dissemination of information on how to use them. In the United States and Northern Ireland,^55^ womxn have been prosecuted for searching for^56^ or ordering abortion pills online for their pregnant daughters. The charges in the US included “providing abortion without a medical license, dispensing drugs without being a pharmacist, assault and endangering the welfare of a child”.^57^ In Uruguay, a number of people were prosecuted for illegally selling misoprostol as the drug is only available within the formal healthcare system and not sold in commercial pharmacies under any indication.^58^ While these legal developments have expanded some abortion access, the shortcomings are significant. Laws that criminalise assistance undermine long-standing practices of feminist solidarity in the form of provision of information or pills that have enabled womxn in many other jurisdictions – from Chile to Ireland to Poland – to navigate some of the most restrictive abortion laws.^20^

Even when the threats do not yield convictions, criminalisation results in further stigma, restriction of information, restriction of access to essential medicines and creates a chilling effect on these practices.^59^ Laws and regulations that apply to SMA may not even be as restrictive as prosecutors or law enforcement’s interpretations suggest. However, they contribute to a widespread social criminalisation whereby health care personnel refuse to provide care or call the police,^60^ NGOs are raided, and activists are harassed,^61^ creating an insidious misperception of abortion as conduct that is criminal at all times. These medically and legally unnecessary restrictions exacerbate inequities and risks by enabling actors who seek to benefit from these restrictive environments. For example, it exposes womxn to financial hardship and social/health risks due to higher prices for MA, counterfeited drugs, misinformation (such as the “abortion reversal” pill) and fake clinics that profit from these restrictive environments.

While simultaneously having an “alegal” approach to realising the human right to abortion and organising trainings for lawyers and legal support for those that encounter legal trouble, feminist collectives resist the cacophony of laws and propose models of abortion advocacy that eschew formal legal mechanisms.^62^

As countries reform their regulations in the context of the COVID-19 pandemic and increasingly adopt models of telemedicine similar to those long used by activists and feminist networks, the legal restrictions become even more cacophonous. For example, the UK’s new guidelines under the COVID-19 pandemic allow womxn to manage their own abortions. Under the new regulations, those requiring abortion care can access a telemedicine consultation with a registered medical practitioner, receive MA pills by post and use them at home.^17^ While acknowledging the differences between the philosophies, power dynamics and models of care underpinning the work of activists and feminist networks, and that of the medical model of care, we point out that the process, active involvement of pregnant persons and the medicines used in telemedicine are not fundamentally different from the care that informal networks such as the *Con las amigas y en la casa* (Chile) and *Las Comadres* (Ecuador) and other feminist groups have been providing for decades. However, the legal risks are significantly different. For example, the care provided for womxn in Northern Ireland^55^ was broadly similar but saw police raids in response. It raises the crucial question of why telemedicine within formally recognised systems is treated differently under the law, compared to those who have provided telehealth care informally for years before. This is particularly stark when the quality of medical care in informal spaces is on a par with formal standards^63,64^ and it is the work of activist networks that has transformed the discourse, technology and science behind the telemedicine model.

When considering womxn’s documented fears of judgement and stigma within formal health systems, feminist abortion care-work caters to their needs across different quality dimensions. Grounded in feminist care models, it is these feminist networks’ years of frontline work, research, innovation and experience that have spurred telemedicine within formal spaces. Yet, it is these feminist models that continue to bear a range of threats and continued questions on the quality, safety and efficacy of the care they provide. This schism demonstrates how the law and criminalisation are tied to the control of abortion, posing the question, “what/who exactly are we legitimising and why?”

Quite differently, the law has not been so zealous in its quest for overregulation of those who disseminate abortion misinformation^65^ or the prosecution of those who harass womxn outside clinics or online; and have – in some contexts – passed laws outright that legally require doctors to tell their patients that “reversal” of MA is an option, going against all scientific evidence.^66^

The SMA landscape as we know it now would not exist without the hard work and risks taken by feminist organisations and networks that have served womxn for decades. Any attempt of regulation must account for their work, include them and must not place further restrictions on their work. For years now, there has been a call for providing abortion in simpler, less medicalised ways that centre womxn’s needs. The pioneering work of hotlines, web-based services and feminist networks demonstrates that this is a real possibility; indeed, it occurs every day within informal and often illegal set-ups the world over. Feminist networks, online counsellors*, acompañantes* and many of the other models proposed by these actors have been instrumental in advancing scientific knowledge and transforming the model of care for abortion. We need to be very vigilant with regard to laws that attempt to regulate SMA, that medicalise it by incorporating a series of unnecessarily burdensome steps or that create vulnerability for the actors involved in safe self-management.

## Conclusion

The history of MA shows us how informal networks have discovered, shared and created the space for SMA to flourish, from Brazilian womxn in the 1980s discovering misoprostol as an abortifacient^67^ to the Abortion Without Borders coalition in 2020.^20^ They have not just enabled the “strategic life choices which are critical for people to live the lives they want”^68^ but have created spaces for innovation, hope and joy. It is these actors who have pushed for the shifts we now see in formal service provision, who have paved the way for better quality care and access. And it is these constellations of actors who, when confronted by a cacophony of laws and increased restrictions, continue to centre pregnant persons and provide/enable SMA against the odds, while risking criminalisation. The constellation of actors, while advancing access to abortion in a range of legal and social conditions, have also fundamentally challenged and altered the meanings of abortion and abortion provision itself: from whose authority and knowledge is valued and centred, to the environments in which abortion is possible, to issuing a broader challenge around how abortion itself is understood and depicted.

Recognising the importance of centring people’s trajectories also means accounting for the constellation of actors that play crucial roles in these experiences. We argue that within SMA research, advocacy and policy, without losing focusing on the “self”, it is important to expand our focus on “management”. The navigation towards safely self-managing abortions includes a huge array of actors, playing key roles, and they should not be made vulnerable by restrictive laws and regulations. These key actors, often working at the frontlines of SMA, are now the targets of politically motivated prosecutors that experiment with a variety of laws to punish not only those who end their own pregnancies but those who support them.^59^ Safety, at the very least, requires accounting for both the outcomes of the procedure and the conditions under which an abortion is obtained.^15^ Thus, we also need to account for the groups of people that are crucial to the process of SMA, who risk their lives, jobs, personal safety, freedom and more by participating in the process of abortion care assistance, procurement and provision.
